# Supplementary material for: Midwifery centers as enabled environments for midwifery: A quasi experimental design assessing women’s birth experiences in three models of care in Bangladesh, before and during covid
Source: PLoS One. 2022 Dec 1;17(12):e0278336. doi: 10.1371/journal.pone.0278336 (PMC9714812; doi:10.1371/journal.pone.0278336)
Supplement: S2 File — (DOCX) [file pone.0278336.s002.docx]

### **S2: Women’s survey**

Woman’s Survey: Respect, Trust, and fear and knowledge of COVID

*1. Info:* Today’s date:___________

*2. Demographic:* Highest level of education completed_______________

*3. Demographic:* Average monthly household income_____________ Number in household_______ (how many live in the house)

4. *Exposure:* Where did you go for prenatal care? Midwifery center UHC Other______________

5. *Exposure:* Where did you give birth? Midwifery center. UHC. Home Other___________

*6. Exposure:* What date did you give birth:_________________

6.a. *Demographic:* How many other times have you given birth?________*(added to calls-June 25 2020)*

“The following questions are about how you felt about your care when you gave birth.”

*RESPECT:* MOR: MOTHERS ON RESPECT INDEX (slightly modified as indicated below)

| Overall while making decisions about my pregnancy or birth care:  (select or circle one answer for each statement) | | Strongly Disagree | Disagree | Somewhat Disagree | Somewhat Agree | Agree | Strongly Agree |
| --- | --- | --- | --- | --- | --- | --- | --- |
| 7 | I was comfortable asking questions | 1 | 2 | 3 | 4 | 5 | 6 |
| 8 | I was comfortable declining care that was offered | 1 | 2 | 3 | 4 | 5 | 6 |
| 9 | I was comfortable accepting the options for care that my midwife recommended | 1 | 2 | 3 | 4 | 5 | 6 |
| 10 | I was pushed into accepting the options my midwife suggested* | 6 | 5 | 4 | 3 | 2 | 1 |
| 11 | I chose the care that I received | 1 | 2 | 3 | 4 | 5 | 6 |
| 12 | My personal preferences were respected | 1 | 2 | 3 | 4 | 5 | 6 |
| 13 | My cultural preferences were respected | 1 | 2 | 3 | 4 | 5 | 6 |
|  | ADD ALL SCORES IN SECTION A: | SECTION A TOTAL SCORE: | | | | | |
| During my pregnancy I felt that I was treated poorly by my midwife because: | | Strongly Disagree | Disagree | Somewhat Disagree | Somewhat Agree | Agree | Strongly Agree |
| 14 | My race, ethnicity, cultural background or language* | 6 | 5 | 4 | 3 | 2 | 1 |
|  | Because of my sexual orientation and/or gender identity (QUESTION REMOVED) | ·· | ·· | ·· | ·· | ·· | ·· |
|  | Because of my health insurance. (QUESTION REMOVED) | ·· | ·· | ·· | ·· | ·· | ·· |
| 15 | A difference of opinion with my midwife about the right care for myself or my baby* | 6 | 5 | 4 | 3 | 2 | 1 |
|  | ADD ALL SCORES IN SECTION B: | SECTION B TOTAL SCORE: | | | | | |
| During my pregnancy I held back from asking questions or discussing my concerns because: | | Strongly Disagree | Disagree | Somewhat Disagree | Somewhat Agree | Agree | Strongly Agree |
| 16 | My midwife seemed rushed* | 6 | 5 | 4 | 3 | 2 | 1 |
| 17 | I wanted care that was different from what my midwife recommended* | 6 | 5 | 4 | 3 | 2 | 1 |
| 18 | I thought my midwife might think I was being difficult* | 6 | 5 | 4 | 3 | 2 | 1 |
|  | ADD ALL SCORES IN SECTION C: | SECTION C TOTAL SCORE: | | | | | |

Vedam, S., Stoll, K., Rubashkin, N., Martin, K., Miller-Vedam, Z., Hayes-Klein, H., & Jolicoeur, G. (2017). The Mothers on Respect (MOR) index: measuring quality, safety, and human rights in childbirth. SSM - Population Health, 3, 201-210. <http://dx.doi.org/10.1016/j.ssmph.2017.01.005>

*TRUST:* **Trust in the public healthcare system**

Medical mistrust Index

|  | Item | Strongly Disagree | Disagree | Neutral | Agree | Strongly Agree |
| --- | --- | --- | --- | --- | --- | --- |
| 19 | You need to be cautious when dealing with health care organizations. | 1 | 2 | 3 | 4 | 5 |
| 20 | Patients are sometimes deceived or mislead by health care organizations. | 1 | 2 | 3 | 4 | 5 |
| 21 | When health care organizations make mistakes, they usually cover it up. | 1 | 2 | 3 | 4 | 5 |
| 22 | Mistakes are common in healthcare organizations. * | 5 | 4 | 3 | 2 | 1 |

Public health care system trust scale *(Health Care provider- Midwife- Individual trust)*

| No | Item | Strongly disagree | Disagree | Neutral | Agree | Strongly Agree |
| --- | --- | --- | --- | --- | --- | --- |
| 23 | I believe my midwife doesn’t tell me everything I need to know. * | 5 | 4 | 3 | 2 | 1 |
| 24 | I believe my midwife will give proper treatment | 1 | 2 | 3 | 4 | 5 |
| 25 | I don’t trust my midwife. * | 5 | 4 | 3 | 2 | 1 |
| 26 | Even if my midwife does a mistake, I still believe in her | 1 | 2 | 3 | 4 | 5 |
| 27 | If I had signs of COVID I wouldn’t tell my midwife* | 5 | 4 | 3 | 2 | 1 |
| 28 | I tell my friends to use my “midwifery center” / “UHC” for health care. | 1 | 2 | 3 | 4 | 5 |

Anand TN, Kutty VR. Development and testing of a scale to measure trust in the public healthcare system. *Indian J Med Ethics.* 2015 Jul-Sep; 12(3): 149-57.

TAKE A BRIEF PAUSE HERE BEFORE THE LAST SECTION, then introduce the last section as questions about her thoughts and feelings around COVID.

**Fear and stigma COVID Questionnaire**

*“When you are pregnant or using health care services, people may treat you differently, or you may feel different about health care because of COVID.”*

*“The next 4 questions are to help us learn what women know about COVID. I can’t help you, but don’t worry, it is just to find out what people know. Please keep your answers short and simple. Just say the first thing that comes to your mind. We will have a chance to talk at the end.”*

*Do NOT read the answers. Listen and circle what she said. IF more is needed, WRITE VERY SHORT ANSWERS, JUST THE KEY WORDS*

29. Knowledge: What causes COVID?

(circle): a Virus. a cold. Air Pollution. ALLAH. I don’t know Other: ___________

30. Knowledge: What are the best ways that people can protect themselves from COVID?

(circle): Social distance Masks. Handwashing Praying. I don’t know Other: ___

31. Knowledge: How does COVID spread from one person to another?

(circle): Coughing. Respiratory droplets. Sneezing. Touching people. Being close to each other Blood. Other: ____________________

32. Info from: How have you learned the most about COVID?

(circle): Husband Family Friends Radio Television Posters Pamphlets …..Midwife Internet. Other:__________________________

33. Knowledge: Do you share your mask with others?

(circle) Never, Sometimes, Always

34. Info: Have you experienced any fever, cough, body aches, sore throat recently?

(circle): YES NO

35. Info: Have you been tested for COVID?

(circle): YES NO *(if yes, was it: positive or negative?)*

| These questions are about some of your thoughts or feelings. | | Strongly disagree  1 | Disagree  2 | Neutral  3 | Agree  4 | Strongly Agree  5 |
| --- | --- | --- | --- | --- | --- | --- |
| 36. | *Fear/stigma:* I think people in my village are afraid of me when I go to the healthcare facility. (i.e. for ANC, birth, PNC) |  |  |  |  |  |
| 37. | *Fear/stigma :* When someone from my village has COVID or dies from COVID/COVID symptoms, but we don’t tell anyone. |  |  |  |  |  |
| The next set of questions is about your experiences with healthcare at the “UHC” or “midwifery center (MC)”. (use which every facility the mother went to) DURING COVID. | | Strongly disagree  1 | Disagree  2 | Neutral  3 | Agree  4 | Strongly Agree  5 |
| 38. | *Barrier:* The lockdown has made getting health care very difficult. |  |  |  |  |  |
| 39. | *Fear /stima :* I am afraid of going to the “UHC”/ “Midwifery Center” because of COVID. |  |  |  |  |  |
| 40. | *Fear/stigma:* I am more afraid to tell health care staff if I feel ill because of COVID. |  |  |  |  |  |
| 41. | *Barrier:* I was dismissed or ignored by healthcare staff when I needed care. circle all staff who treated you this way: Doctor, Nurse, Midwife, Other |  |  |  |  |  |

42. *info:* Is there anything more you want to share about the impact of COVID on your life? __________________________________________________________________________________

Revised from: James et al. BMC Public Health (2020) 20:182. https://doi.org/10.1186/s12889-020-8279-7
