# Supplementary material for: Midwifery centers as enabled environments for midwifery: A quasi experimental design assessing women’s birth experiences in three models of care in Bangladesh, before and during covid
Source: PLoS One. 2022 Dec 1;17(12):e0278336. doi: 10.1371/journal.pone.0278336 (PMC9714812; doi:10.1371/journal.pone.0278336)
Supplement: S3 File — (DOCX) [file pone.0278336.s003.docx]

### **S3: Women participant response rate by model of care**

|  | | **FEM**  **Model**  **(n= 363)** | | **MAM**  **Model**  **(n= 312)** | | **NOM**  **Model**  **(n= 515)** | | | **Totals**  **(n=1,190)** |
| --- | --- | --- | --- | --- | --- | --- | --- | --- | --- |
| **Pre COVID participants**  **(Jan-March 2020)** | Total # women w/SVDs  w/MW/nurse/nurseMW  Jan-March 2020 | 321 | | 416 | | 931 | | | 1,668 |
|  | # eligible women (w/mobile**)** | 280  (87·2%) | | 296  (71·2%) | | 415  (44·6%) | | | 991  (59·4%) |
|  | Total # pre COVID Participants | 207 | | 190 | | 318 | | | 715 |
|  | Response rate Pre COVID | 73·9% | | 64·2% | | 76·6% | | | 72·1% |
| **COVID participants (April-June 2020)** | Total # women w/SVDs  w/MW/nurse/nurseMW  April-June 2020 | 260 | | 294 | | 403 | | | 957 |
|  | # eligible women (w/mobile) | 213  (82%) | | 141  (48%) | | 254  (63%) | | | 608  (63·5%) |
|  | Total # COVID Participants | 156 | | 122 | | 197 | | | 475 |
|  | Response rate COVID | 73·2% | | 86·5% | | 77·6% | | | 78·1% |
| **By facility** | | **Mir** | **Jain.** | **Sav** | **Sri.** | **Rang** | **Kula** | **Gaff** | **··** |
| # of women participants by facility | | 122 | 241 | 60 | 252 | 395 | 90 | 30 |  |
| % of MOC participants by facility | | 33·6% | 66·4% | 19·2 % | 80·8% | 76·7% | 17·5% | 5·8% |  |
| **Total # Study Participants** | | 363 | | 312 | | 515 | | | 1190 |
| Total # of women w/SVD  Jan-June 2021  w/MW/nurses/nurse-MW | | 581 | | 710 | | 1,334 | | | 2,625 |
| Total % response rate of all women w/SVD w/& wo mobile phone | | 62·5% | | 44% | | 38·6% | | | 45·3% |
| **Total # eligible women for study period** | | 493 (84·5%) | | 437(61·5%) | | 669 (50%) | | | 1,599  (60·9%) |
| **Total % response rate of eligible women** | | 73·6% | | 71·4% | | 77% | | | 74·4% |

SVD= Spontaneous vaginal delivery, MW= Midwife, ANC= antenatal care, MOC= Model of care

Facilities: Mir= Mirpur MC, Dhaka, Jain=Jaintapur MC, Sylhet, Sav= Savar UHC, Dhaka, Sri= Srimongol UHC, Sylhet, Rang=Rangpur MCH, Rangpur , Kula,=Kulaura UHC, Sylhet, Gaff= Gaffargaow UCH, Mymensingh.
