# Supplementary material for: Midwifery centers as enabled environments for midwifery: A quasi experimental design assessing women’s birth experiences in three models of care in Bangladesh, before and during covid
Source: PLoS One. 2022 Dec 1;17(12):e0278336. doi: 10.1371/journal.pone.0278336 (PMC9714812; doi:10.1371/journal.pone.0278336)
Supplement: S4 File — (DOCX) [file pone.0278336.s004.docx]

### **S4: Facility data by model of care**

|  | **FEM** | | **MAM** | | **NOM** | | |
| --- | --- | --- | --- | --- | --- | --- | --- |
| **Facility name and location** | Mirpur MC, Dhaka | Jaintapur MC, Sylhet | Savar UHC,  Dhaka | Sreemangal UHC, Sylhet | Rangpur MCH, Rangpur | Kulaura UHC, Sylhet | Gofargaon UHC, Mymensingh |
| **Total SVD* Jan-June 2019** | 237 | 279 | 308 | 1440 | 2717 | 541 | 843 |
| **Number of SVD Jan-June 2019 w/MW* or nurses** | 237 | 279 | 115 | 650 | 1373 | 238 | 422 |
| **Total SVD* Jan-June 2020** | 199 | 382 | 278 | 1,429 | 2,166 | 375 | 688 |
| **Number of SVD Jan-June 2020 w/MW* or nurses** | 199 | 382 | 125 | 585 | 865 | 178 | 291 |
| **% change in total # of SVD* from same time previous year** | -16% | +37% | -10% | -1% | -20.3% | -30.7% | -18.4% |
| **Annual data Births/year** | 390 (est) | 765 (est) | 2015: 299 | 2015:1539 35 vavd | 2015 : 5,550 110 vavd | 2015: 2,250 | 2015 : 712 |
| **# SVD*** | 390 (est) | 765 (est) | 157 | 1305 | 3441 | 2250 | 712 |
| **#C/S*** | 0 | 0 | 142 | 199 | 1999 | 0 | 0 |
| **C/S* Rate** | 0 | 0 | 47.5% | 12.9% | 36% | 0% | 0% |
| **Total Beds in the facility** | 2 | 2 | 50 | 50 | 1000 | 50 | 50 |

SVD= Spontaneous vaginal delivery, C/S= cesarean section, MW= midwife, VAVD= vacuum assisted vaginal delivery

FEM= Fully enabled midwifery, MAM= Midwifery and Medicine, NoM= No Midwifery

The national drop in facility-based births during COVID was reflected in our data as well, with an overall drop of 20% in participants during COVID when compared to the pre-pandemic period, with one exception. The Jaintapur midwifery center experienced a 37% increase in birth volume compared to 2019. All other facilities experienced a decline in birth volume compared to the same time period in 2019.
