# Supplementary material for: Midwifery centers as enabled environments for midwifery: A quasi experimental design assessing women’s birth experiences in three models of care in Bangladesh, before and during covid
Source: PLoS One. 2022 Dec 1;17(12):e0278336. doi: 10.1371/journal.pone.0278336 (PMC9714812; doi:10.1371/journal.pone.0278336)
Supplement: S5 File — (DOCX) [file pone.0278336.s005.docx]

### **S5: Description of participants in the different care models**

| **Groups:** | **FEM: n= 363** | | **MAM: n=312** | | **NoM: n=515** | | **Total study population: n= 1190** | |
| --- | --- | --- | --- | --- | --- | --- | --- | --- |
| **Study population pre and during COVID** | Pre: 207 | C19: 156 | Pre: 190 | C19: 122 | Pre: 318 | C19: 197 | Pre: 715 | C19: 475 |
| **Mean yrs of Education** | Educ: 6·51 yrs | | Educ: 7·20 yrs | | Educ: 8·82 yrs | | Educ: 7·69 yrs | |
| **SD for education** | 3·5 yrs | | 4·21 yrs | | 4·52 yrs | | 4·28 yrs | |
| **Mean years of education pre and during covid** | Pre:  6·24 yrs | C19:  6·86 yrs | Pre:  8·1 yrs | C19: 5·79 | Pre: 8·78 | C19: 8·89 | Pre: 7·89 | C19: 7·43 |
| **Average monthly Income (in Taka)** | Income: 10,806 ($130·22) | | Income: 11,402 ($137·37) | | Income: 11,434 ($137·76) | | Income: 11,234 ($135·35) | |
| **SD for Income** | 6,367 ($76·71) | | 5,599 ($67·46) | | 5,840 ($70·36) | | 5,946 ($71·64) | |
| **Average Monthly Income (in Taka)** | 10,213 | 11,593 | 11,787 | 10,803 | 11,809 | 10,833 | .. | .. |
| **Mean Parity** | Parity: 1·58 | | Parity: 0·814 | | Parity: 1·10 | | Parity: 1·17 | |
| **SD for parity** | 1·15 | | 0·95 | | 1·18 | | 1·15 | |
| **Nulliparous** | 67 (18%) | | 145 (46%) | | 194 (38%) | | 406 (34%) | |
| **Multiparous** | 296 (82%) | | 167 (54%) | | 321 (62%) | | 784 (66%) | |

FEM= Fully enabled midwifery MAM= Midwifery and medicine NoM= No midwifery
