# Supplementary material for: Midwifery centers as enabled environments for midwifery: A quasi experimental design assessing women’s birth experiences in three models of care in Bangladesh, before and during covid
Source: PLoS One. 2022 Dec 1;17(12):e0278336. doi: 10.1371/journal.pone.0278336 (PMC9714812; doi:10.1371/journal.pone.0278336)
Supplement: S6 File — (DOCX) [file pone.0278336.s006.docx]

### **S6: Group and covariates significance and coefficient**

|  | **Respect Pre COVID** | | **Respect during COVID** | | **Trust Pre COVID** | | **Trust during COVID** | | **Covid Fear during COVID** | |
| --- | --- | --- | --- | --- | --- | --- | --- | --- | --- | --- |
|  | **p** | **Coefficient** | **p** | **Coefficient** | **p** | **Coefficient** | **p** | **Coefficient** | **p** | **Coefficient** |
| **Model of care** | <0·001 | Intercept FEM:  5·66e+01  MAM:  8·52e-01 NoM:  -4·32e+ 00 | <0·001 | Intercept  FEM:  54·62  MAM:  -9·29  NoM:  -5·27 | <0·001 | Intercept FEM:  3·59e+01 MAM:  -1·42e+ 00 NoM:  -3·32e+ 00 | <0·001 | Intercept FEM:  3·50e+01 MAM:  **-**3·22e+ 00 NoM:  -3·25e+ 00 | <0·001 | Intercept FEM:  7.13e+00 MAM:  2.59e+00 NoM**:**  5·25e-01 |
| **Educ.** | 0·191 | -8·45e-02 | 0·109 | 0·11 | 0·754 | -1·33e-02 | 0·002 | 9·46e-02 | 0·97 | -6·43e-03 |
| **Parity** | 0·223 | 3·15e-01 | 0·005 | 0·69 | 0·215 | 1·63e-01 | 0·283 | 1·15e-01 | 0·40 | -8·05e-02 |
| **Income** | 0·119 | 6·68e-05 | 0·011 | 0·0001 | 0·094 | 3·64e-05 | 0·009 | 6·82e-05 | 0·73 | 6·56e-06 |
