# Supplementary material for: Midwifery centers as enabled environments for midwifery: A quasi experimental design assessing women’s birth experiences in three models of care in Bangladesh, before and during covid
Source: PLoS One. 2022 Dec 1;17(12):e0278336. doi: 10.1371/journal.pone.0278336 (PMC9714812; doi:10.1371/journal.pone.0278336)

### **S7: Outcomes by facility**

| **Groups:** | **FEM: n=363** | | **MAM: n=312** | | **NoM: n=515** | | |
| --- | --- | --- | --- | --- | --- | --- | --- |
| **Study population pre and during COVID** | **Pre: 207** | **C19: 156** | **Pre: 190** | **C19: 122** | **Pre: 318** | **C19: 197** | |
| **Facility name and location** | Mirpur MC, Dhaka | Jaintapur MC, Sylhet | Savar UHC, Dhaka | Sreemangal UHC, Sylhet | Rangpur MCH, Rangpur | Kulaura UHC, Sylhet | Gofargaon UHC, Mymensingh |
| **# of participants by facility** | 122 | 241 | 60 | 252 | 395 | 90 | 30 |
| **Mean total respect** | 57·63 | | 53·01 | | 52·66 | | |
| **Mean total respect by facility** | 57·30 | 57·80 | 58·63 | 51·67 | 51·87 | 56·93 | 50·27 |
| **Significance between facilities** | p=0·398 | | p<0·001 | | ·· | p<0·001 | p=0·016 |
| **CI** | -1·64, 0·66 | | 5·92, 8·00 | | ·· | -6·30, -3·83 | 0·29, 2·89 |
| **Mean Trust total** | 36·57 | | 34·28 | | 33·27 | | |
| **Mean trust total by facility** | 36·01 | 36·85 | 36·42 | 33·78 | 32·90 | 34·11 | 35·60 |
| **Significance between facilities** | p=0·013 | | p<0·001 | | ·· | p=0·0016 | p<0·001 |
| **CI** | -1·51, -0·17 | | 2·10, 3·18 | | ·· | -1·96, -0·46 | -3·39,-2·014 |
| **Mean COVID fear total** | 8·61 | | 8·44 | | 8·57 | | |
| **Mean Covid fear total by facility** | 8·77 | 8·53 | 8·05 | 8·54 | 8·67 | 6·71 | 12·86 |
| **Significance between facilities** | p=0·392 | | p=0·067 | | ·· | p<0·001 | p<0·001 |
| **CI** | -0·31, 0·79 | | -1·01, 0·35 | | ·· | 1·39, 2·52 | -4·70, -3·69 |

When trying to understand the reason behind the decline in women’s experience of respectful care and trust, and rise in COVID fear during the pandemic in the MAM model, we considered midwives working without support and the culture of the facilities itself.

Could some of the differences between outcomes and facilities be from more impersonal care in larger facilities? The larger facilities in MAM and NoM provided the majority of data for each of those models. In MAM, the facility at Seermongol had the lowest mean for women’s trust compared to Savar (the other facility in MAM). Savar had only 60 participants. Could a smaller facility provide more responsive care? If we had the majority of participants from smaller facilities in the MAM model, would the outcome results have been better? Both of the MAM facilities had a drop in their reported mean for women’s trust during the pandemic, although it was minimal in the smaller Savar facility. All other facilities, in other models, had either stable means (no significant change) or a slight non-significant rise in their mean for women’s trust during the pandemic.(See Figure 1 for results of trust by facility)

#### S7 Fig.1: Trust Mean (sd) by facility pre/pandemic period

**NoM facilities**

Goffor: n=23/3

35.56 (1.58)/36 (1)

Kular: n=60/30

33.63 (2.85)/35.07 (3.19) Rangpur: n=230/165

32.89 (3.97)/32.91 (4.13)

**MAM facilities**

Savar: n=47/13

36.49 (1.35)/36.15 (2.27) Seermong: n=143/109 34.50 (2.60)/32.84 (2.95)

**FEM facilities**

Mirpur: n=68/54

36.17 (3.56)/35.81 (2.72) Sylhet: n=139/102

36.70 (2.74)/37.06 (2.61)


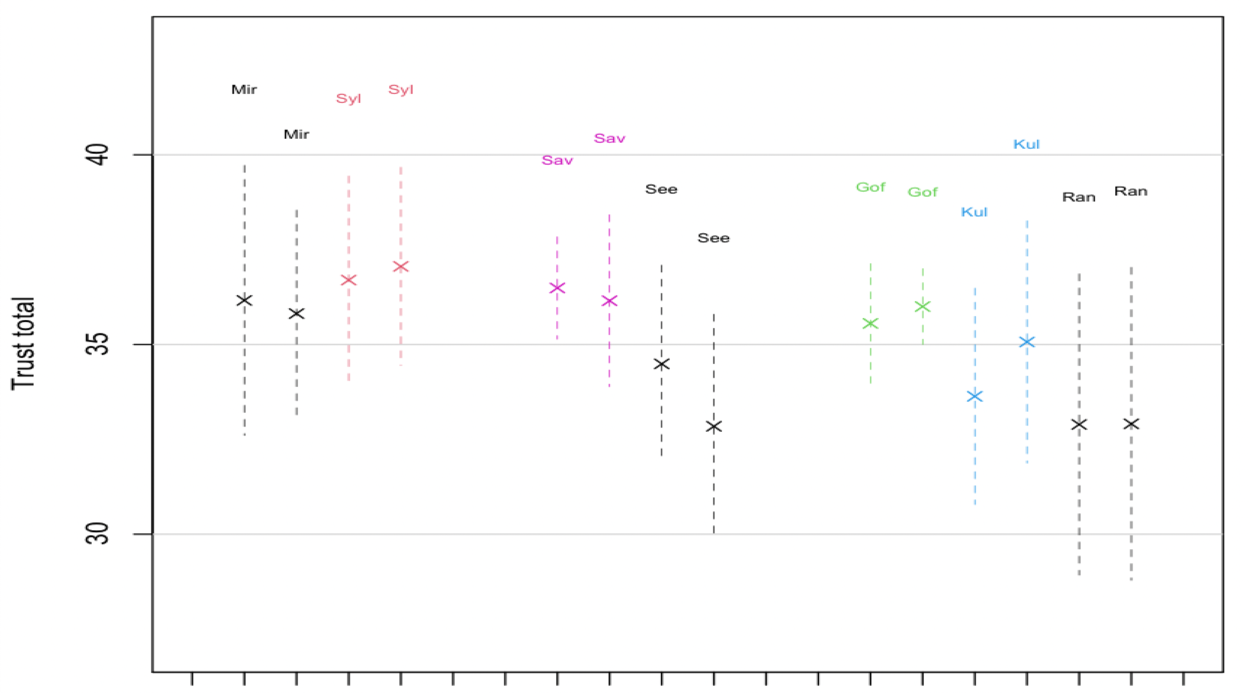


Both facilities in MAM had significant drops in their mean results for the experience of respectful care during the pandemic as well. Savar actually had the highest mean for respectful care in the pre-pandemic period when compared to the other facilities.

Two facilities in the NoM model, Rangpur and Gofargaon, reported low means of respectful care pre and in the pandemic period compared to the other models. In fact the small facility of Gofargaon had the lowest result pre-pandemic. But it was women who gave birth at the largest facility, Rangpur, who shared stories of babies dying. (See Figure 2 for results of respectful care by facility)

The data collectors felt the women’s poor experiences of care in Seermongol were related to its poor reputation, and stated, “people in the community don’t trust the care there”. Were the women biased against care at Seermongol? But the mean for respectful care was low in the pre-pandemic period, and dropped the most during the pandemic period, perhaps reflecting not just the community’s sense of trust but the woman’s experience of respectful care.

#### S7 Fig.2: Respect Mean (sd) by facility pre and pandemic period

**NoM facilities**

Goffor: n=23/3

50.11 (2.65)/51.67 (1.53) Kular: n=60/30

56.7 (4.70)/57.4 (2.42) Rangpur: n=230/165 52.24 (9.24)/51.34 (8.75)

**FEM facilities**

Mirpur: n=68/54

57.65 (5.97)/56.87 (5.5)

Sylhet: n=139/102

57.18 (3.47)/58.64 (4.60)

**MAM facilities**

Savar: n=47/13

59 (1.76)/57.30 (4.13) Seermong: n=143/109 55.19 (4.48)/47.06 (6.0)


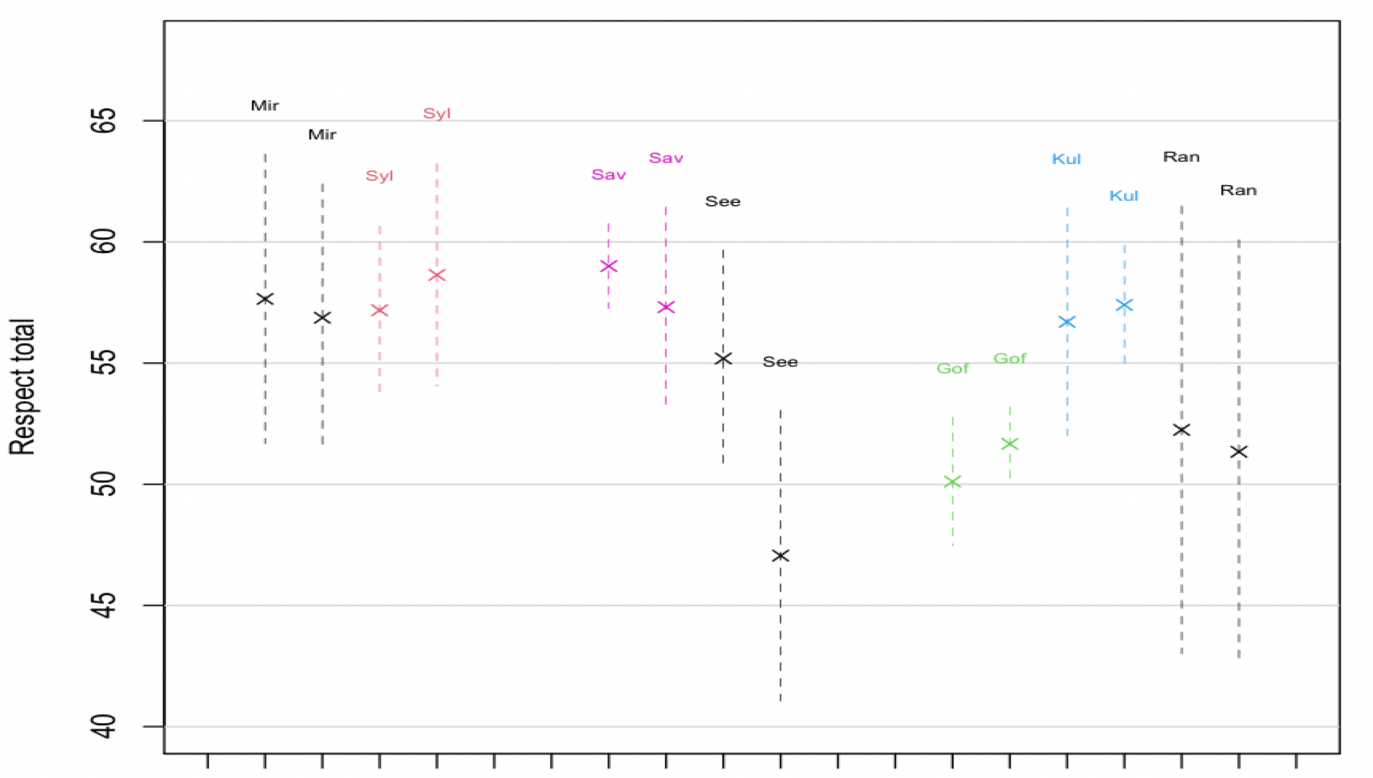

Supplement: S7 File — (DOCX) [file pone.0278336.s007.docx]
